# Re-Evaluation of Imaging Methods of Reactive Oxygen and Nitrogen Species in Plants and Fungi: Influence of Cell Wall Composition

**DOI:** 10.3389/fphys.2017.00826

**Published:** 2017-10-24

**Authors:** Michaela Sedlářová, Lenka Luhová

**Affiliations:** ^1^Department of Botany, Faculty of Science, Palacký University Olomouc, Olomouc, Czechia; ^2^Department of Biochemistry, Faculty of Science, Palacký University Olomouc, Olomouc, Czechia

**Keywords:** confocal microscopy, fluorescent probes, reactive oxygen species, reactive nitrogen species, cell wall

## Abstract

Developmental transitions and stress reactions in both eukaryotes and prokaryotes are tightly linked with fast and localized modifications in concentrations of reactive oxygen and nitrogen species (ROS and RNS). Fluorescent microscopic analyses are widely applied to detect localized production of ROS and RNS *in vivo*. In this mini-review we discuss the biological characteristics of studied material (cell wall, extracellular matrix, and tissue complexity) and its handling (concentration of probes, effect of pressure, and higher temperature) which influence results of histochemical staining with “classical” fluorochromes. Future perspectives of ROS and RNS imaging with newly designed probes are briefly outlined.

## Past and presence of fluorescent probes for localization of reactive oxygen and nitrogen species

Reactive oxygen species (ROS) are generated and scavenged over the whole life span of all known types of aerobic organisms. In plants and fungi production of ROS, together with reactive nitrogen species (RNS), has been linked with almost all developmental processes from germination through reproduction until cell death (Asada, 2006; Blokhina and Fagerstedt, 2010). ROS and RNS represent two classes of highly reactive signaling compounds indispensable also for stress reactions to extreme environmental factors, pathogens, or injuries (Wojtaszek, 1997; Qiao et al., 2014; Del Río, 2015; Dietz et al., 2016; Sedlářová et al., 2016; Raja et al., 2017). In spite of extensive studies, metabolism of both ROS forms, i.e., molecular (H_2_O_2_, hydrogen peroxide; ^1^O_2_, singlet oxygen) and free radicals (${\text{O}}_{2•}^{-}$, superoxide anion; OH_•_, hydroxyl radical; HO_2•_, perhydroxy radical; RO_•_, alkoxy radicals), and RNS (^·^NO, nitric oxide; ONOO^−^, peroxynitrite; and others) still has not been completely understood. Quite recently, peroxynitrite (formed upon NO reaction with superoxide anion) was shown as a positive regulator of plant cell signaling by tyrosine nitration in proteins (Vandelle and Delledonne, 2011) and tightly linked to necrotrophic phase of oomycete pathogenesis (Arasimowicz-Jelonek et al., 2016). ROS and NO-mediated signaling is tightly connected with molecules influencing normal ontogeny, acclimation, and pathophysiology, including multiple hormones, enzymes, and genes (Gill and Tuteja, 2010; León et al., 2014; Nie et al., 2015; Saxena et al., 2016; Raja et al., 2017). Timing of generation, degradation, and diffusion of ROS and RNS within different cellular compartments have therefore attracted attention in many model organisms (Del Río, 2015; Considine et al., 2017). Cross-talk of ROS and RNS has been pointed out also in peroxisomes (Corpas et al., 2017) and signal transduction to other organelles, e.g., mitochondria, Golgi, and endoplasmic reticulum, has been shown (Wanders et al., 2016).

Methods for ROS/RNS detection in plant material based on histochemical staining, e.g., with 3,3′-diaminobenzidine (DAB) for hydrogen peroxide (Thordal-Christensen et al., 1997) or nitro blue tetrazolium chloride (NBT) for superoxide (Jabs et al., 1996), are still being applied for stereomicroscopy and light microscopy, esp. in *Arabidopsis* research. Cell-permeable fluorescence-based probes were subsequently introduced to detect tiny real-time changes in ROS and RNS levels within relevant cellular compartments, e.g., DCF DA and DHDCF DA for detection of ROS (Kehrer and Paraidathathu, 1992; Hempel et al., 1999), DAF-2 DA and DAF-FM DA for NO (Kojima et al., 1999; Lombardo et al., 2006), or SOSG for singlet oxygen (Flors et al., 2006; Kim et al., 2013). A wide range of ROS and NO targeted fluorescent probes has been marketed but some of the most commonly used ones were found to suffer from low selectivity and specificity toward the analyte (e.g., DHDCF DA) or from photosensitization during incubation and microscopy (e.g., SOSG). In order to minimize artifacts, a sample staining in dark and visualization by (multiphoton) confocal microscopy has been advised. Nevertheless, fluorochromes able to cross plasma membrane (e.g., in diacetate form) which can be loaded into cells just by placing the samples (cells, tissues) into a solution of the dye significantly simplified ROS and RNS *in vivo* monitoring and enabled expansion of these techniques within plant science community. Considering the use of proper controls (e.g., ROS/RNS donors for positive controls, and ROS/RNS scavengers for negative ones), proper sample washing, keeping constant time of staining/scanning within a set of experiments, using optimal pH and turgor pressure can contribute to obtaining of correct results. Still it should be emphasized that histochemical staining and subsequent microscopic detection cannot be used for accurate ROS/RNS quantification but the combinations of several different analytical methods can give more reliable estimation of their intracellular levels (Gupta and Igamberdiev, 2013).

**Figure 1:**
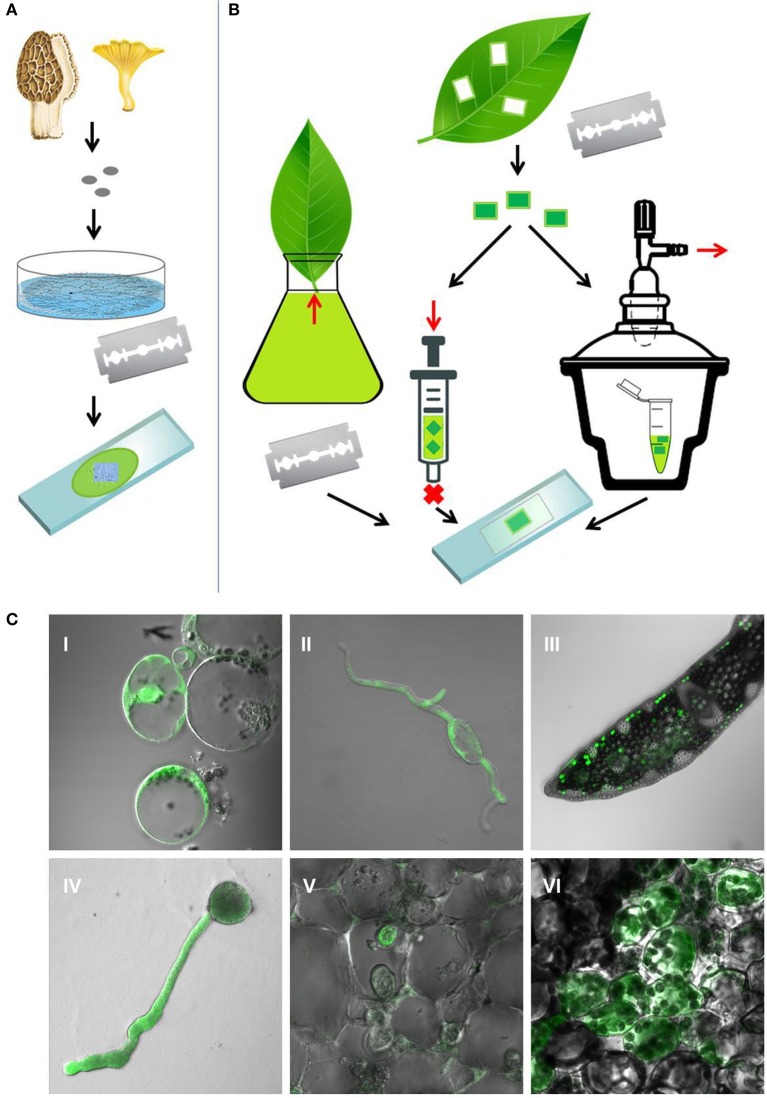
Histochemical detection of ROS and NO with fluorescent probes. **(A)** ROS and RNS in fungi and oomycetes grown on agar can be stained and visualized directly on the medium. **(B)** ROS and RNS in plant tissues and phytopathogenic oomycetes or fungi can be stained by up-loading the probes to excised leaves by xylem transport or to small pieces of tissue using syringe or vacuum infiltration. **(C)** ROS and RNS detection (green signal) by confocal microscopy in different samples: **(I–III)** ROS detection by DHDCF DA in **(I)** cucumber protoplast 4 h after release (10 μM, 10 min), **(II)** 8 h germinated conidia of *Morchella conica* (20 μM, 15 min), **(III)** in mesophyll cells of date palm leaf cross section during drought stress (20 μM, 10 min); **(IV,V)** NO production localized by DAF-FM DA in **(IV)** 2 h germinated cucumber pollen (10 μM, 30 min) and **(V)** haustoria of *Plasmopara halstedii* infecting sunflower stem mesophyll cells (20 μM, 30 min); **(VI)** singlet oxygen visualization with SOSG during mechanical injury of mesophyll cells of *Arabidopsis thaliana* cv. Columbia-0 (50 μM, 30 min) (*M. Sedlářová*).

Optimization of staining procedures for different photosynthetic and fungal organisms in our laboratory showed that results of ROS/RNS imaging in multicellular biological matrices are significantly influenced by the feasibility of material infiltration with the applied probes (Figure 1). Current studies unveiled cell wall (CW) as a dynamic structure able to adapt to various conditions of growth, development, and environmental stresses; together with plasma membrane and periplasmic space, it regulates the flow of molecules into and out of the cell (Lesage and Bussey, 2006). The relative composition of polysaccharides, phenolic compounds, and proteins in CW varies among species and cell types, and changes with their developmental stage (Popper et al., 2011, 2014; Ochoa-Villarreal et al., 2012). In addition, stress factors induce CW reinforcement, such as deposition of lignin or callose in plant-pathogen interactions (Prats et al., 2008; Sedlářová et al., 2011; Miedes et al., 2014). Similarly, materials deposited either intercellularly or in tissue exterior (e.g., cutin and suberin, polyesters which function as permeability barriers to the movement of water) influence the penetration rate of used fluorescence probes. Our extensive experience, based on optimizing incubation conditions for different materials, combined with literary data resulted in Table 1 which summarizes cell wall composition in photosynthetic and fungal organisms together with comparison of concentrations used for ROS/RNS imaging with three commonly used probes (DHDCF DA, DAF-FM DA, and SOSG). Optimal experimental conditions (incubation time, temperature, probe concentration) differ among various model phototrophic organisms (higher plants, algae, and cyanobacteria), fungi and “fungi-like” organisms (oomycetes; Table 1). Although, the unicellular structures [protoplasts (Figure 1CI), pollen (Figure 1CIV), green algae, and thin-walled spores (Figure 1CII)] can be stained easily in general the probe concentration must be increased and incubation time prolonged for cyanobacteria, which are characterized by higher cross-linking of polysaccharides in the cell wall and production of external mucoid sheath (Hoiczyk and Hansel, 2000). For unicellular cyanobacterium *Synechocystis*, widely used photosynthetic model, the concentration of SOSG was increased from commonly used 50 μM up to 250 μM together with incubation temperature increased from room temperature to 37°C (Sinha et al., 2012). Relatively easy staining and imaging can be achieved on agar media (Figure 1A) for germinating fungi (Figure 1CII) and some oomycetes but also for plant pollen (Figure 1CIV) and small seeds. Higher concentrations of probes are advisable for plant tissues (Table 1; Figures 1CIII,V,VI). Excised leaves uptake the probes by xylem transport but longer periods of such incubations are inappropriate for most fluorochromes (Figure 1B). Natural openings like stomata (which represent ~1% of leaf blade epidermal cells, 50–300/mm^2^), hydatodes (at leaf edge), or nectaria (in flowers) can enhance the introduction of fluorochromes into the living tissues of above-ground plant organs. The fluorochromes uptake in multicellular organs can thus be enhanced by increased external or decreased internal pressure, e.g., by syringe or vacuum infiltration, respectively (Figure 1B). Moreover, cutting tissue into pieces significantly increases penetration rates (Figures 1CV,VI) but several layers of mechanically injured cells on the cutting edge must be omitted from the evaluation (Prasad et al., 2017).

**Table 1:** Comparison of cell wall (CW) properties in photosynthetic organisms, fungi, and oomycetes with regards to used concentrations of selected ROS and RNS fluorescent probes.

**Group of organisms**	**CW layout and thickness**	**CW chemical composition**	**External stratum/permeability barrier**	**References**	**Concentration of widely used fluorochromes**		
					**DHDCF DA^a^**	**DAF-FM DA^b^**	**SOSG^c^**
Higher plants	Up to three layers = primary, internally formed secondary CW, middle lamella (outermost); 0.1 to several μm	Polysaccharides (cellulose + hemicelluloses + pectin); Lignin; Proteins (enzymes, expansins); pollen—sporopollenin, rhamnogalacturonan II	Cuticle = cutin and wax (external to CW); suberin (Casparian strips in root endodermis and cork cells in bark); in grasses—microscopic Si crystals	Popper et al., 2011, 2014; Ochoa-Villarreal et al., 2012; Miedes et al., 2014;	Whole tissues (leaves, roots) 10–20 μM	20–40 μM	50–260 μM
					Sections 10–20 μM	10–20 μM	50 μM
					Protoplasts, pollen 5–10 μM	10 μM	30–50 μM
Algae	Multilayered, variable in different taxonomic groups; up to 0.5 μm	Polysaccharides (cellulose + others—depending on taxonomic group: mannans, xylans, alginic acid, or sulfonated polysaccharides (agarose, carrageenan, porphyran, furcelleran and funoran) or a variety of glycoproteins (Volvocales) or both); Sporopollenin; Phlorotannins in brown algae; Diatoms synthesize CW known as frustules or valves from orthosilicic acid	Extracellular matrix—sheath or envelope of mucilage outside the cell made of exopolysaccharides	Popper et al., 2011, 2014; Mine et al., 2016	Single-celled species 10 μMFilamentous algae 20 μMDiatoms 10 μM	10 μM10–20 μM10 μM	50 μM50–100 μM260 μM
Oomycetes	Monolayer, up to 0.3 μm; oospore—multi-layered, up to 2 μm	Polysaccharides (cellulose and glucans); proteins; CW includes hydroxyproline, which is not found in fungal CW	Extracellular matrix in tissue-infecting species	Grenville-Briggs et al., 2013; Mélida et al., 2013	Conidia 10 μMIntercellular mycelium 10–20 μM	10 μM10–20 μM	50 μM50–100 μM
Fungi	Bilayered—secondary CW is external to primary, width 0.05–0.4 μm; Spores—multi-layered, thick up to 10 μm; special morphology of septa in hyphae	Chitin (in Ascomycota and Basidiomycota), or chitosan (Zygomycota); Glucans; Proteins (enzymes, structural proteins esp. mannoproteins); in spores—melanin, sporopollenin	Outer layer or capsule with mannans and glucans (namely in pathogens); Many hyphal and spore surfaces covered with hydrophobins; Glomalin (glycoprotein abundantly secreted in arbuscular mycorrhizal fungi)	Ruiz-Herrera, 1992; Lesage and Bussey, 2006; Latgé, 2007; Erwig and Gow, 2016	Spores, mycelium 10–20 μM (to be increased if mycelium grown in agar)	10–40 μM	50–100 μM
Cyanobacteria	Multilayered, structure similar to G- bacteria; width 10 nm in unicellular species; 15–35 nm in filamentous (extremely thick in *Oscillatoria princeps* = 700 nm)	Peptidoglycan and outer membrane composed of fibrilar lipopolysaccharides, carotenoids, and porins	Slime coat, capsule, mucoid sheath	Hoiczyk and Hansel, 2000	20–40 μM (to be increased in filamentous species with thick CW)	40–50 μM	50–250 μM

a DHDCF DA = 2′,7′-dichlorodihydrofluorescein diacetate; max. λex = 498 nm/λex = 522 nm; oxidized by hydroperoxides, other ROS and peroxynitrite; standard incubation time 10–15 min (Hempel et al., 1999; Petřivalský et al., 2012);

b DAF-FM DA = 4-amino-5-(N-methylamino)-2′,7′-difluorofluorescein diacetate; max. λex = 495 nm/λex = 515 nm; oxidized by NO_2_; standard incubation time 30 min (Kojima et al., 1999; Lombardo et al., 2006; Sedlářová et al., 2011);

c *SOSG = Singlet Oxygen Sensor Green, a dyad composed of fluorescein and anthracene moieties; max. λex = 508 nm/λex = 530 nm; oxidized by singlet oxygen; standard incubation time 30 min (Flors et al., 2006; Sinha et al., 2012; Kim et al., 2013; Prasad et al., 2017)*.

## Dawn of reliable ROS and RNS imaging?

Recognized drawbacks of commercially available fluorescence probes for ROS and RNS detection initiated a quest for improved tools to measure more accurately the differential *in vivo* patterns of ROS and RNS abundance within plant organs and meristems. Newly synthesized probes with increased specificity and improved photostability have been reported, such as Aarhus Sensor Green preferable to SOSG for singlet oxygen (Pedersen et al., 2014), but these are for various reasons of limited availability to users. Therefore, the need for further development of improved probes that can image individual endogenous ROS and RNS still continues. Recently, a new family of o-hydroxyamino-triarylpyrylium salts-based probes for NO detection was reported (Beltrán et al., 2014). A new fluorescent probe ContPY1 was prepared for investigations of hydrogen peroxide and tested in *Arabidopsis*, both on cultured cells and on leaves (Ledoux et al., 2013). Also, a single fluorescent probe, capable of simultaneous monitoring of both NO and H_2_O_2_ endogenously produced in living macrophages (Yuan et al., 2012) was synthesized. However, similarly to genetically encoded fluorescence proteins applicable for ROS monitoring (Schmitt et al., 2014) or immuno-spin traping (Mason, 2016), it has not yet been successfully applied to plant research.

Fluorescein derivatives have become replaced in animal ROS and RNS research by more specific molecular probes based on nanoparticles or redox-sensitive fluorescent proteins (for review see Guo et al., 2014; Peteu et al., 2014). As an example, the entirely new probe PAM-BN-PB (composed of three functional parts: phenanthroimidazole, benzonitrile, and phenyl boronate) was designed to detect H_2_O_2_ with good selectivity based on intramolecular charge transfer (Chen et al., 2017), and tested on human and animal cells and *in vitro*. However, the “classical fluorescent probes” based mainly on diaminofluorescein derivatives, still represent important tools to study ROS and RNS in plant science (Nie et al., 2015; Figure 1C). This can be partly attributed to more demanding protocols due to presence of CW and other extracellular matrices (Table 1) influencing the uptake of “new generation” probes. Encapsulating fluorescent probes into nanoparticles was reported to improve their stability, such as in peroxalate nanoprobe undergoing a three-component chemiluminescence reaction between H_2_O_2_, peroxalate esters, and fluorescent dyes as published for *in vivo* imaging of H_2_O_2_ in mouse model (Lee et al., 2007). Near-IR probes have been lately incorporated into polymeric micelles modified with animal cell-penetrating peptides, esp. for peroxynitrite imaging experiments (Tian et al., 2011). However, (nano)micelles uptake by fusion with the plasma membrane is hindered in plant and fungal cells and up-to-date protocols for the cell wall removal exert excessive oxidative stress to the plant cells (Petřivalský et al., 2012).

Although, a plethora of ROS and RNS sensing molecules have been designed, just a part of them has been confirmed experimentally to be suitable for ROS and RNS *in vivo* monitoring. The situation resembles a “population bottle-neck”; only a reduced number of protocols are applicable to ROS and RNS microscopy in plant and fungal models and thus these few remain fixed in routine practice for a substantial period. With increasing knowledge on the importance of localized and tiny intracellular redox fluctuations the quantitative and spatio-temporal analysis of ROS and RNS levels in plant and fungal cells is still highly challenging.

## Author contributions

MS prepared manuscript based on long-lasting discussions and joint experiments with LL, it was approved and widely discussed by both authors.

### Conflict of interest statement

The authors declare that the research was conducted in the absence of any commercial or financial relationships that could be construed as a potential conflict of interest.

## Abbreviations

CW: cell wall
DHDCF DA: 2′,7′-dichlorodihydrofluorescein diacetate
DAF-FM DA: 4-amino-5-(N-methylamino)-2′,7′-difluorofluorescein diacetate
RNS: reactive nitrogen species
ROS: reactive oxygen species
SOSG: Singlet Oxygen Sensor Green.

## References

[B1] Arasimowicz-Jelonek M. Floryszak-Wieczorek J. Izbianska K. Gzyl J. Jelonek T. (2016). Implication of peroxynitrite in defence responses of potato to *Phytophthora infestans*. Plant Pathol. 65, 754–766. 10.1111/ppa.12471

[B2] Asada K. (2006). Production and scavenging of reactive oxygen species in chloroplasts and their functions. Plant Physiol. 141, 391–396. 10.1104/pp.106.082040 16760493 PMC1475469

[B3] Beltrán A. Isabel Burguete M. Abanades D. R. Perez-Sala D. Luis S. V. Galindo F. (2014). Turn-on fluorescent probes for nitric oxide sensing based on the ortho-hydroxyamino structure showing no interference with dehydroascorbic acid. Chem. Commun. 50, 3579–3581. 10.1039/c3cc49555h 24567953

[B4] Blokhina O. Fagerstedt K. V. (2010). Reactive oxygen species and nitric oxide in plant mitochondria: origin and redundant regulatory systems. Physiol. Plant. 138, 447–462. 10.1111/j.1399-3054.2009.01340.x 20059731

[B5] Chen Y. Shi X. Lu Z. Wang X. Wang Z. (2017). Fluorescent probe for hydrogen peroxide *in vivo* based on the modulation of intramolecular charge transfer. Anal. Chem. 89, 5278–5284. 10.1021/acs.analchem.6b04810 28415838

[B6] Considine M. J. Diaz-Vivancos P. Kerchev P. Signorelli S. Agudelo-Romero P. Gibbs D. J.. (2017). Learning to breathe: developmental phase transitions in oxygen status. Trends Plant Sci. 22, 140–153. 10.1016/j.tplants.2016.11.013 27986423

[B7] Corpas F. J. Barroso J. B. Palma J. M. Rodriguez-Ruiza M. (2017). Peroxisomes: a nitro-oxidative cocktail. Redox Biol. 11, 535–542. 10.1016/j.redox.2016.12.033 28092771 PMC5238456

[B8] Del Río L. A. (2015). ROS and RNS in plant physiology: an overview. J. Exp. Bot. 66, 2827–2837. 10.1093/jxb/erv099 25873662

[B9] Dietz K.-J. Mittler R. Noctor G. (2016). Recent progress in understanding the role of reactive oxygen species in plant cell signaling. Plant Physiol. 171, 1535–1539. 10.1104/pp.16.00938 27385820 PMC4936595

[B10] Erwig L. P. Gow N. A. (2016). Interactions of fungal pathogens with phagocytes. Nat. Rev. Microbiol. 14, 163–176. 10.1038/nrmicro.2015.21 26853116

[B11] Flors C. Fryer M. J. Waring J. Reeder B. Bechtold U. Mullineaux P. M.. (2006). Imaging the production of singlet oxygen *in vivo* using a new fluorescent sensor, Singlet oxygen sensor green. J. Exp. Bot. 57, 1725–1734. 10.1093/jxb/erj181 16595576

[B12] Gill S. S. Tuteja N. (2010). Reactive oxygen species and antioxidant machinery in abiotic stress tolerance in crop plants. Plant Physiol. Biochem. 48, 909–930. 10.1016/j.plaphy.2010.08.016 20870416

[B13] Grenville-Briggs L. J. Horner N. R. Phillips A. J. Beakes G. W. van West P. (2013). A family of small tyrosine rich proteins is essential for oogonial and oospore cell wall development of the mycoparasitic oomycete. Fungal Biol. 117, 163–172. 10.1016/j.funbio.2013.01.001 23537873

[B14] Guo H. Aleyasin H. Dickinson B. C. Haskew-Layton R. E. Ratan R. R. (2014). Recent advances in hydrogen peroxide imaging for biological applications. Cell Biosci. 4:64. 10.1186/2045-3701-4-64 25400906 PMC4232666

[B15] Gupta K. J. Igamberdiev A. U. (2013). Recommendations of using at least two different methods for measuring NO. Front. Plant Sci. 4:58. 10.3389/fpls.2013.00058 23520440 PMC3603275

[B16] Hempel S. L. Buettner G. R. O'Malley Y. Q. Wessels D. A. Flaherty D. M. (1999). Dihydrofluorescein diacetate is superior for detecting intracellular oxidants: comparison with 2′,7′-dichlorodihydrofluorescein diacetate, 5(and 6)-carboxy-2′,7′-dichlorodihydrofluorescein diacetate, and dihydrorhodamine 123. Free Radic. Biol. Med. 27, 146–159. 10.1016/S0891-5849(99)00061-1 10443931

[B17] Hoiczyk E. Hansel A. (2000). Cyanobacterial cell walls: news from an unusual prokaryotic envelope. J. Bacteriol. 182, 1191–1199. 10.1128/JB.182.5.1191-1199.2000 10671437 PMC94402

[B18] Jabs T. Dietrich R. A. Dangl J. L. (1996). Initiation of runaway cell death in an Arabidopsis mutant by extracellular superoxide. Science 273, 1853–1856. 10.1126/science.273.5283.1853 8791589

[B19] Kehrer J. P. Paraidathathu T. (1992). The use of fluorescent probes to assess oxidative processes in isolated-perfused rat heart tissue. Free Radic. Res. Commun. 16, 217–225. 10.3109/10715769209049175 1505782

[B20] Kim S. Fujitsuka M. Majima T. (2013). Photochemistry of singlet oxygen sensor green. J. Phys. Chem. B 117, 13985–13992. 10.1021/jp406638g 24111566

[B21] Kojima H. Urano Y. Kikuchi K. Higuchi T. Hirata Y. Nagano T. (1999). Fluorescent indicators for imaging nitric oxide production. Angew. Chem. Int. Ed. Engl. 38, 3209–3212. 10.1002/(SICI)1521-3773(19991102)38:21<3209::AID-ANIE3209>3.0.CO;2-6 10556905

[B22] Latgé J. P. (2007). The cell wall: a carbohydrate armour for the fungal cell. Mol. Microbiol. 66, 279–290. 10.1111/j.1365-2958.2007.05872.x 17854405

[B23] Ledoux Q. Veys P. Van Cutsem P. Mauro S. Lucaccioni F. Marko I. E. (2013). Validation of the boronate sensor ContPY1 as a specific probe for fluorescent detection of hydrogen peroxide in plants. Plant Signal. Behav. 8:e26827. 10.4161/psb.26827 24169206 PMC4091588

[B24] Lee D. Khaja S. Velasquez-Castano J. C. Dasari M. Sun C. Petros J.. (2007). *In vivo* imaging of hydrogen peroxide with chemiluminescent nanoparticles. Nat. Mater. 6, 765–769. 10.1038/nmat1983 17704780

[B25] León J. Castillo M. C. Coego A. Lozano-Juste J. Mir R. (2014). Diverse functional interactions between nitric oxide and abscisic acid in plant development and responses to stress. J. Exp. Bot. 65, 907–921. 10.1093/jxb/ert454 24371253

[B26] Lesage G. Bussey H. (2006). Cell wall assembly in *Saccharomyces cerevisiae*. Microbiol. Mol. Biol. Rev. 70, 317–343. 10.1128/MMBR.00038-05 16760306 PMC1489534

[B27] Lombardo M. C. Graziano M. Polacco J. C. Lamattina L. (2006). Nitric oxide functions as a positive regulator of root hair development. Plant Signal. Behav. 1, 28–33. 10.4161/psb.1.1.2398 19521473 PMC2633697

[B28] Mason R. P. (2016). Imaging free radicals in organelles, cells, tissue, and *in vivo* with immuno-spin trapping. Redox Biol. 8, 422–429. 10.1016/j.redox.2016.04.003 27203617 PMC4878322

[B29] Mélida H. Sandoval-Sierra J. V. Diéguez-Uribeondo J. Bulone V. (2013). Analyses of extracellular carbohydrates in oomycetes unveil the existence of three different cell wall types. Eukaryot. Cell 12, 194–203. 10.1128/EC.00288-12 23204192 PMC3571302

[B30] Miedes E. Vanholme R. Boerjan W. Molina A. (2014). The role of the secondary cell wall in plant resistance to pathogens. Front. Plant Sci. 5:358. 10.3389/fpls.2014.00358 25161657 PMC4122179

[B31] Mine I. Yamasaki T. Sekida S. Okuda K. (2016). Measurement of cell wall thickness in the giant-celled xanthophycean alga Vaucheria frigida. Cytologia 81, 225–230. 10.1508/cytologia.81.225

[B32] Nie S. Yue H. Zhou J. Xing D. (2015). Mitochondrial-derived reactive oxygen species play a vital role in the salicylic acid signaling pathway in *Arabidopsis thaliana*. PLoS ONE 10:e0119853. 10.1371/journal.pone.0119853 25811367 PMC4374720

[B33] Ochoa-Villarreal M. Aispuro-Hernández E. Vargas-Arispuro I. Martínez-Téllez M. Á. (2012). Plant cell wall polymers: function, structure and biological activity of their derivatives, in Polymerization, ed Gomes A. D. S. (Rijeka: InTech), 63–86.

[B34] Pedersen S. K. Holmehave J. Blaikie F. H. Gollmer A. Breitenbach T. Jensen H. H.. (2014). Aarhus sensor green: a fluorescent probe for singlet oxygen. J. Org. Chem. 79, 3079–3087. 10.1021/jo500219y 24605923

[B35] Peteu S. F. Boukherroub R. Szunerits S. (2014). Nitro-oxidative species biosensing: challenges and advances with focus on peroxynitrite quantification. Biosens. Bioelectron. 58, 359–373. 10.1016/j.bios.2014.02.025 24681525

[B36] Petřivalský M. Vaníčková P. Ryzí M. Navrátilová B. Piterková J. Sedlářová M. (2012). The effects of reactive nitrogen and oxygen species on regeneration and growth of cucumber cells from isolated protoplasts. Plant Cell Tissue Organ Cult. 108, 237–249. 10.1007/s11240-011-0035-3

[B37] Popper Z. A. Michel G. Hervé C. Domozych D. S. Willats W. G. Tuohy M. G.. (2011). Evolution and diversity of plant cell walls: from algae to flowering plants. Annu. Rev. Plant Biol. 62, 567–590. 10.1146/annurev-arplant-042110-103809 21351878

[B38] Popper Z. A. Ralet M. C. Domozych D. S. (2014). Plant and algal cell walls: diversity and functionality. Ann. Bot. 114, 1043–1048. 10.1093/aob/mcu214 25453142 PMC4195566

[B39] Prasad A. Sedlářová M. Kale R. Pospíšil P. (2017). Lipoxygenase in singlet oxygen generation as a response to wounding: *in vivo* imaging in *Arabidopsis thaliana*. Sci. Rep. 7:9831. 10.1038/s41598-017-09758-1 28851974 PMC5575249

[B40] Prats E. Carver T. L. Mur L. A. (2008). Pathogen-derived nitric oxide influences formation of the appressorium infection structure in the phytopathogenic fungus *Blumeria graminis*. Res. Microbiol. 159, 476–480. 10.1016/j.resmic.2008.04.001 18554873

[B41] Qiao W. Li C. Fan L.-M. (2014). Cross-talk between nitric oxide and hydrogen peroxide in plant responses to abiotic stresses. Environ. Exp. Bot. 100, 84–93. 10.1016/j.envexpbot.2013.12.014

[B42] Raja V. Majeed U. Kang H. Andrabi K. I. John R. (2017). Abiotic stress: interplay between ROS, hormones and MAPKs. Environ. Exp. Bot. 137, 142–157. 10.1016/j.envexpbot.2017.02.010

[B43] Ruiz-Herrera J. (1992). Fungal Cell Wall: Structure, Synthesis and Assembly. Boca Raton, FL: CRC Press.

[B44] Saxena I. Srikanth S. Chen Z. (2016). Cross talk between H2O2 and interacting signal molecules under plant stress response. Front. Plant Sci. 7:570. 10.3389/fpls.2016.00570 27200043 PMC4848386

[B45] Schmitt F.-J. Renger G. Friedrich T. Kreslavski V. D. Zharmukhamedov S. K. Los D. A.. (2014). Reactive oxygen species: re-evaluation of generation, monitoring and role in stress-signaling in phototrophic organisms. Biochim. Biophys. Acta 1837, 835–848. 10.1016/j.bbabio.2014.02.005 24530357

[B46] Sedlářová M. Kubienová L. Drábková Trojanová Z. Luhová L. Lebeda A. Petřivalský M. (2016). The role of nitric oxide in development and pathogenesis of biotrophic phytopathogens – downy and powdery mildews. Adv. Bot. Res. 77, 263–283. 10.1016/bs.abr.2015.10.002

[B47] Sedlářová M. Petřivalský M. Piterková J. Luhová L. Kočírová J. Lebeda A. (2011). Influence of nitric oxide and reactive oxygen species on development of lettuce downy mildew in *Lactuca* spp. Eur. J. Plant Pathol. 129, 267–280. 10.1007/s10658-010-9626-9

[B48] Sinha R. K. Komenda J. Knoppová J. Sedlářová M. Pospíšil P. (2012). Small CAB-like proteins prevent formation of singlet oxygen in the damaged Photosystem II complex of the cyanobacterium *Synechocystis* sp. PCC 6803. Plant Cell Environ. 35, 806–818. 10.1111/j.1365-3040.2011.02454.x 22070528

[B49] Thordal-Christensen H. Zhang Z. Wei Y. Collinge D. B. (1997). Subcellular localization of H2O2 in plants. H2O2 accumulation in papillae and hypersensitive response during the barley-powdery mildew interaction. Plant J. 11, 1187–1194. 10.1046/j.1365-313X.1997.11061187.x

[B50] Tian J. Chen H. Zhuo L. Xie Y. Li N. Tang B. (2011). A highly selective, cell-permeable fluorescent nanoprobe for ratiometric detection and imaging of peroxynitrite in living cells. Chem. Eur. J. 17, 6626–6634. 10.1002/chem.201100148 21590826

[B51] Vandelle E. Delledonne M. (2011). Peroxynitrite formation and function in plants. Plant Sci. 181, 534–539. 10.1016/j.plantsci.2011.05.002 21893249

[B52] Wanders R. J. Waterham H. R. Ferdinandusse S. (2016). Metabolic interplay between peroxisomes and other subcellular organelles including mitochondria and the endoplasmic reticulum. Front. Cell Dev. Biol. 3:83. 10.3389/fcell.2015.00083 26858947 PMC4729952

[B53] Wojtaszek P. (1997). Oxidative burst: an early plant response to pathogen infection. Biochem. J. 322(Pt 3), 681–692. 10.1042/bj3220681 9148737 PMC1218243

[B54] Yuan L. Lin W. Xie Y. Chen B. Zhu S. (2012). Single fluorescent probe responds to H2O2, NO, and H2O2/NO with three different sets of fluorescence signals. J. Am. Chem. Soc. 134, 1305–1315. 10.1021/ja2100577 22148503

